# Interpersonal Conflicts and Development of Self-Esteem from Adolescence to Mid-Adulthood. A 26-Year Follow-Up

**DOI:** 10.1371/journal.pone.0164942

**Published:** 2016-10-18

**Authors:** Olli Kiviruusu, Noora Berg, Taina Huurre, Hillevi Aro, Mauri Marttunen, Ari Haukkala

**Affiliations:** 1 Department of Health, National Institute for Health and Welfare, Helsinki, Finland; 2 Department of Health and Social Welfare, Unit of School Social Work, City of Vantaa, Finland; 3 School of Health Sciences, University of Tampere, Tampere, Finland; 4 University of Helsinki and Helsinki University Hospital, Adolescent Psychiatry, Helsinki, Finland; 5 Department of Social Research, University of Helsinki, Helsinki, Finland; Central Institute of Mental Health, GERMANY

## Abstract

This study investigated the association between interpersonal conflicts and the trajectory of self-esteem from adolescence to mid-adulthood. The directionality of effects between self-esteem and interpersonal conflicts was also studied. Participants of a Finnish cohort study in 1983 at age 16 (N = 2194) were followed up at ages 22 (N = 1656), 32 (N = 1471) and 42 (N = 1334) using postal questionnaires. Measures covered self-esteem and interpersonal conflicts including, conflicts with parents, friends, colleagues, superiors, partners, break-ups with girl/boyfriends, and divorces. Participants were grouped using latent profile analysis to those having “consistently low”, “decreasing”, or “increasing” number of interpersonal conflicts from adolescence to adulthood. Analyses were done using latent growth curve models and autoregressive cross-lagged models. Among both females and males the self-esteem growth trajectory was most favorable in the group with a consistently low number of interpersonal conflicts. Compared to the low group, the group with a decreasing number of interpersonal conflicts had a self-esteem trajectory that started and remained at a lower level throughout the study period. The group with an increasing number of interpersonal conflicts had a significantly slower self-esteem growth rate compared to the other groups, and also the lowest self-esteem level at the end of the study period. Cross-lagged autoregressive models indicated small, but significant lagged effects from low self-esteem to later interpersonal conflicts, although only among males. There were no effects to the opposite direction among either gender. Our results show that those reporting more and an increasing number of interpersonal conflicts have a lower and more slowly developing self-esteem trajectory from adolescence to mid-adulthood. While the result was expected, it does not seem to imply an effect from interpersonal conflicts to low self-esteem. Rather, if anything, our results seem to suggest that those with low self-esteem are more prone to later interpersonal conflicts.

## Introduction

Global self-esteem, as the general evaluative attitude that we take towards ourselves, is very much a social psychological construct: it is rooted in social interaction and it develops hand in hand with our interpersonal relations [1,2]. Early social psychologists have pointed out the looking glass nature of our self-view [3,4], thus emphasizing the importance of other people’s views to our self-concept. Indeed, individuals derive their self-worth from the feedbacks they receive from significant others [5]. The process of developing self-esteem is life-long and a key role in it is played by interpersonal relationships. It begins in early childhood, where a secure attachment to parents forms a basis for a healthy self-esteem [5], but continues in adolescence, first to include peer relations [6], and in adulthood to comprise the interpersonal relationships of our different social contexts and roles [7]. The idea that self-esteem is due to social relationships is taken to its extreme in the sociometer theory of self-esteem [1,8], which considers self-esteem as a mere gauge of our relational value, a sociometer, indicating the degree we are accepted or rejected in our social world.

Interpersonal conflicts and quarrels are part of our daily lives and to a certain extent they can be thought of as a “normal thing”. However, it might be that some individuals experience these types of problems and conflicts more often than others. This may be, for example, because of issues relating to personality or inadequate social skills [9,10]. However, problems and conflicts may also accumulate in certain life situations or circumstances, leading to vicious circles [11]. Whatever the reason, they represent collisions, and sometimes actual disruptions, in our social fabric; they might be indications of our changing relational value, already altered or under serious negotiations before changing. And to the extent that interpersonal conflicts mean less acceptance and more rejection, they are likely to have a negative effect on our feelings of ourselves, thus lowering self-esteem [1].

However, the causal relationship or directionality of effects between self-esteem and interpersonal conflicts can go both ways. The sociometer theory of self-esteem would suggest that interpersonal problems, conflicts and break-ups are likely indications of low acceptance and a heightened risk of rejection, consequently leading to low relational value, i.e. low self-esteem [1,8]. The fact that interpersonal rejection, devaluation and exclusion lead to lower self-esteem has also been shown in the many experimental studies testing the tenets of the sociometer theory; for a list of studies see [1]. While the effect from interpersonal conflicts to low self-esteem seems thus evident, there are also results supporting the view that the effect goes from low self-esteem to interpersonal conflicts. In their test of social-adaptation theory, Kahle and colleagues [12] found support only for the effect in the direction from low self-esteem to interpersonal problems, but no support for the effect in the opposite direction. Moreover, Donnellan and colleagues [13] found a robust relation between low self-esteem and externalizing problems, such as antisocial behavior among adolescents and young adults, suggesting one possible mechanism between low self-esteem and interpersonal conflicts. Furthermore, Baldwin [7] has suggested that there is a form of behavioral confirmation through which self-esteem shapes relationship quality in that “an individual with low self-esteem, who anticipates that others will be critical and rejecting, somehow manages to produce exactly this kind of response from interaction partners”. Thus, while self-esteem is the result of our social interactions, it also provides a filter through which we view and respond to the behavior of others. In the light of these studies, there seem to be both theoretical reasons and empirical findings to support the view that effects between self-esteem and interpersonal conflicts are reciprocal.

In the recent years there has been an increasing number of studies on self-esteem development using individual change trajectories covering the years from adolescence to adulthood [14–17], as well as up to old ages [18–20]. These studies have indicated a relatively consistent developmental pattern where self-esteem increases from adolescence to young adulthood, continues to increase, although more slowly, during mid-adulthood and turns to a decreasing trajectory around the 50s or 60s [18–20]. Some of these studies have also addressed the question of how interpersonal factors shape the self-esteem trajectory during the life course. In their cohort sequential study between ages 25 and 104, Orth and colleagues [18] found marital status, social support and relationship satisfaction to have no effect on the level or shape of the self-esteem trajectory. However in a more recent study relationship satisfaction was related to a more positive life-span trajectory of self-esteem [20]. Regarding years from adolescence to young adulthood, Birkeland and colleagues [16] found that those in the high self-esteem trajectory group had better relationships with parents, although not with friends, compared to those in the chronically low self-esteem group. Also Greene and Way [15], in their longitudinal study from 13 to 20 years, found that positive changes in family and friend support were associated with improvements in self-esteem. The only study that had directly addressed the effect of interpersonal conflicts on the self-esteem trajectory is the study by Galambos and colleagues [14] among young adults followed from age 18 to 25 years: they found conflicts with parents to be associated with a lower initial level, but not with the slope of the self-esteem trajectory. Thus, the results regarding the effect of interpersonal relationship variables on self-esteem development have been quite mixed, while the measures and studied developmental periods have been quite different as well. Furthermore, only one study has addressed the effect of interpersonal conflicts on self-esteem trajectory directly and it covered only a relatively short developmental period of seven years.

### The present study

Good self-esteem can be regarded as an essential component of mental health, and research indicates that it predicts a variety of other important life outcomes [19,21]. It is important to gain knowledge of the correlates of self-esteem, especially those relevant for self-esteem development during the life course. While conflicts and problems in interpersonal relations can be considered as the type of phenomena at the heart of the social psychological construct of self-esteem, they have gained only little research attention in studies on the life course development of self-esteem. Using a sample which at baseline comprised practically a full age cohort of one Finnish city, the present study addresses this question by exploring the association between self-esteem and interpersonal conflicts from adolescence at age 16 to mid-adulthood at age 42 years. We have shown earlier that in this study cohort, and in line with other studies, self-esteem increases from adolescence to adulthood [17]. The present study aims at developing our understanding of the correlates of this developmental trajectory of self-esteem. More specifically, we address two main research questions:

Q1) Is the developmental trajectory of self-esteem from adolescence to mid-adulthood associated with longitudinal patterns of interpersonal conflicts? For example, if there is a group of individuals with a pattern showing an increasing amount of interpersonal conflicts during the study period, is this increasing pattern reflected also in their developmental trajectory of self-esteem? Based on theories and earlier research results indicating the important role of interpersonal relations to self-esteem and its development, we expect high and increasing levels of interpersonal conflicts to be associated with a lower and more slowly growing self-esteem trajectory.

While the first study question might reveal associations between self-esteem development and longitudinal patterns of interpersonal conflicts, it cannot say anything about the directionality of the effects between self-esteem and interpersonal conflicts. Thus, our second study question addresses this issue:

Q2) Does self-esteem affect later interpersonal conflicts, or is it rather that conflicts in interpersonal relationships have an effect on later self-esteem; or are these effects bidirectional, going simultaneously in both directions? Based on theoretical reasons and previous research findings, we are inclined to expect that the effects between self-esteem and interpersonal conflicts are bidirectional.

In addition to the primary study questions, we are also interested to see whether there are any gender differences in these phenomena. Firstly, this is because we know from previous research that males have a higher self-esteem level than females [22]; this gender difference was shown to be quite pronounced also in this cohort, although the growth rate of the self-esteem trajectory was somewhat faster among females [17]. Secondly, it has been suggested that interpersonal events are more common among females and also that they are more vulnerable in the face of these events [11].

## Method

### Ethics statement

The study protocol has been approved by the Ethics Committee of Tampere University Hospital and the Ethics Committee of the National Institute for Health and Welfare, Finland. Participants were informed of the purposes of the study and that participation was voluntary and they indicated their consent by answering the survey questionnaire.

### Subjects

The original target population included all Finnish-speaking ninth-grade pupils attending secondary school in the spring of 1983 in Tampere, an industrial university city in southern Finland with 166 000 inhabitants at that time. In 1983, a total of 2194 pupils (96.7% of the target population) completed a questionnaire during school hours. Of the participants, 1071 were females and 1123 were males, the mean age was 15.9 (SD 0.3) years. Participants in the 1983 baseline study were followed up by postal questionnaires in 1989 (N = 1656, 75.5%), 1999 (N = 1471, 67.0%) and 2009 (N = 1334, 60.8%) when they were 22, 32 and 42 years of age, respectively.

Of the participants, 87.6% (N = 1922) participated in at least one follow-up survey after the baseline, 70.1% (N = 1538) in at least two and 45.6% (N = 1001) in all three follow-ups. Only 12.4% (N = 272) of the participants did not participate in any of the three follow-ups after the baseline assessment. Attrition was examined by correlating the study variables at age 16 (see measures below) with the number of waves of participation (range 1–4). On average there were 3.0 responses per participant, the number being higher in females than males (3.3 vs. 2.8, p<0.001). However, response was not related to self-esteem or number of interpersonal conflicts at age 16 either among females or males. Attrition has also been studied earlier in this study project by Eerola et al. [23] regarding the first three study waves. In that study, male gender and poorer school performance at age 16 (self-reported grade point average from the previous school report) were the strongest predictors of non-response. This missingness-related information was taken into account in the present analyses where appropriate (see statistical analyses section).

### Measures

#### Self-esteem

The measure of self-esteem consisted of seven statements (on a five-point scale) of self-worth resembling those used in Rosenberg's measure [24,25]. The statements were "I believe in myself and in my possibilities", "I wish I was different from how I am" (reversed), "I suffer from feelings of inferiority" (reversed), "I think I have many good qualities", "I feel I lack self-confidence" (reversed), "I am capable of doing the same as others", and "I am often dissatisfied with myself" (reversed). The self-esteem score was calculated as the mean of item scores (theoretical range 1–5). Cronbach's alphas ranged from 0.80 to 0.89 in the different panel waves.

#### Interpersonal conflicts

As part of a life events checklist the subjects were asked whether they had experienced problems or increased conflicts in their interpersonal relationships (0 = no, 1 = yes) during the past 12 months [26,27]. The checklist was modified for each follow-up questionnaire to accommodate the life stage in question. In the measure of interpersonal conflicts the following events/categories were included: increased conflicts with mother, father, teacher/superior, classmates, friends, colleagues/fellow students; increased conflicts in intimate relationship; break-up with girl-/boyfriend, separation/divorce. There were five such events/problem categories in the checklist at age 16, seven at age 32 and eight at ages 22 and 42 years (for conflict categories in each follow-up questionnaire, see S1 Table). These events/categories were summed up in each panel wave to form an index of interpersonal conflicts and the right tails were cut so that the highest score was "4 or more" (range 0–4) in all waves of the study.

### Statistical analyses

Analyses were made using Mplus 7.1 software [28] and IBM SPSS Statistics 22.0.

To address the first study question regarding the association between longitudinal patterns of interpersonal conflicts and self-esteem development, the analyses were conducted in three steps. First, the participants were grouped according to their longitudinal profiles of interpersonal conflicts using longitudinal latent profile analysis (LLPA) [29], which is a form of latent profile analysis (LPA) [30] applied for longitudinal data. Latent profile analysis is a finite mixture model method that can be used to identify homogenous unobserved groups or profiles based on observed variables. Since LLPA has no a priori assumptions on the general pattern or functional form of the growth [29], it is also suitable in situations where the observed variables (measures) are not exactly the same between panel waves. In the analyses, interpersonal conflict index variables were mean centered (with a mean of zero) in each wave of the study so that the group profiles reflect deviations from the mean level of the interpersonal conflict index in a given study wave. The primary aim of the LLPA was to find a small number (2–4) of easily interpretable groups to be used in the subsequent analyses. The statistical criteria used to determine the best solution, i.e. the number of profiles, were the Bayesian Information Criteria (BIC) and the Bootstrapped Likelihood Ratio Test (BLRT), both of which have been indicated to perform well in deciding the number of classes in mixture modeling [31]. However, as the identification of latent profiles per se was not the primary focus in the present study, emphasis was also placed on the usefulness of the group solution for the subsequent analyses, i.e. on distinctively different profiles with large enough group sizes. After determining the best group solution, cases were assigned to the latent profile groups according to their most likely profile group membership.

In the second step, the trajectory of self-esteem was analyzed with latent growth models (LGM). In a linear LGM, the change trajectory is modeled by means of two latent variables: the intercept (level) and slope (growth rate), and the time loadings of the slope factor are fixed to represent the linearly growing time between the panel waves, in this case 0, 0.6, 1.6, and 2.6 (in tens of years) for the consecutive waves, respectively. Non-linear trajectories can be modeled using higher order growth factors or by estimating some loadings of the slope factor freely from the data [32].

In the third step, differences in the self-esteem growth trajectories between interpersonal profile groups were compared using multigroup analyses. For this purpose, one multiple group LGM of self-esteem development was specified, comprising all latent interpersonal conflict profile groups for both females and males. Group differences in self-esteem development were analyzed in terms of initial levels (age 16), slopes, and end levels (age 42) of self-esteem and tested with chi-square (χ^2^) change statistic, comparing models with and without the parameter of interest constrained to be the same between groups (two groups at a time). Gender differences in the effects of interpersonal conflict profiles on self-esteem growth factors were analyzed in additional models, comprising two profile groups at a time and regressing the self-esteem growth factors on the profile group variable.

The second study question, the directionality of effects between self-esteem and interpersonal conflicts was analyzed using an autoregressive cross-lagged (ARCL) model. The ARCL model can be used to analyze the direction of effects between constructs, since effects are prospectively tested and autoregressive effects are controlled for. The interpretation of a significant cross-lagged effect is that the earlier measure of construct B (B_t-1_) prospectively predicts the later measure of construct A (A_t_) over and above the autoregressive effect of the earlier measure of A (A_t-1_). One multiple group (females, males) ARCL model was specified, and gender differences were tested using chi-square (χ^2^) change statistic.

To deal with missing values due to attrition, the full information maximum likelihood (FIML) estimation method was used, which produces less biased and more reliable results compared with conventional methods of dealing with missing data, such as listwise or pairwise deletion [33,34]. As attrition was related to school achievement at age 16 [23], it was used as an auxiliary, missingness-related variable in the analyses to increase the plausibility of the missing at random (MAR) assumption. (While attrition was also related to gender, it could not be used in a similar manner, as it was already part of the analysis model, i.e. multigroup by gender.)

All analyses were made first without any adjustments and then with adjustments for parental divorce before age 16 (yes/no) and parental socioeconomic status at age 16 (blue collar/lower white collar/upper white collar): as the results remained essentially the same, the original estimates without adjustments are presented. Model fit was assessed by the Tucker-Lewis index (TLI), the comparative fit index (CFI), and the root mean square error of approximation (RMSEA). TLI and CFI values ≥ 0.95 and RMSEA values ≤ 0.06 were considered to indicate a good fit to the data [35].

## Results

The descriptive statistics of the study variables are given in Table 1. Observed means of self-esteem increased from age 16 to 32 years, but remained the same between ages 32 and 42. Males had higher self-esteem in all study waves, whereas females reported more interpersonal conflicts at ages 16 and 42 years.

**Table 1:** Descriptive statistics of the study variables by gender.

	Females (N = 1071)		Males (N = 1123)		Gender difference
Variable	N	Mean (SD)	N	Mean (SD)	p (t-test)
Self-esteem^a^ at age 16	1067	3.47 (0.67)	1105	3.83 (0.64)	<0.001
Self-esteem^a^ at age 22	886	3.56 (0.71)	764	3.90 (0.67)	<0.001
Self-esteem^a^ at age 32	803	3.86 (0.74)	663	4.10 (0.71)	<0.001
Self-esteem^a^ at age 42	733	3.88 (0.75)	597	4.15 (0.67)	<0.001
Interpersonal conflicts^b^ at age 16	1069	0.78 (0.98)	1113	0.60 (0.89)	<0.001
Interpersonal conflicts^b^ at age 22	888	1.16 (1.25)	764	1.10 (1.18)	0.377
Interpersonal conflicts^b^ at age 32	804	1.04 (1.18)	665	0.95 (1.12)	0.149
Interpersonal conflicts^b^ at age 42	732	1.01 (1.17)	599	0.79 (1.08)	0.001

^a^ Range 1–5

^b^ Range 0–4

**Fig 1:**
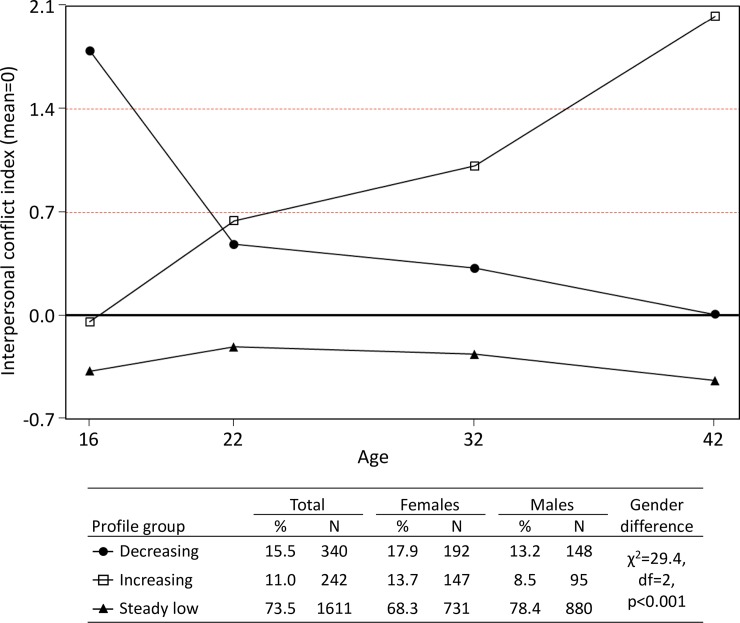
Longitudinal profiles of interpersonal conflicts, three-class LLPA solution. Observed means of interpersonal conflict indexes (for total sample) and group sizes according to most likely class membership.

To identify latent profiles of interpersonal conflicts from adolescence to mid-adulthood, LLPA with two-, three- and four-class solutions were run. Of these, the four-class solution had problems in model estimation and had to be discarded. When comparing the two and three-group solutions, both the BIC values (18877.0 for the three-class, 19176.9 for the two-class model) and the BLRT statistic (p<0.001) indicated that the three-class solution was better in terms of statistical criteria. As the three-group solution was also interpretationally better in terms of producing groups with distinctively different profiles while maintaining large enough groups sizes, it was chosen for the subsequent analyses. Entropy of the three-group solution was 0.787. The interpersonal conflict profiles of the selected three-group solution are presented in Fig 1. The largest profile group was the “steady low” (73.5%), constantly reporting interpersonal conflicts below average, and with the lowest levels of conflicts of the three groups throughout the study period. The other two profile groups were clearly different from this “normative” profile and from each other: individuals in the “decreasing” group (15.5%) reported on average 1.8 interpersonal conflict categories above the total mean level at age 16, albeit this gradually declined during the study period (more rapidly between ages 16 and 22) and the group ended up at the mean level of interpersonal conflicts at age 42. The “increasing” (11.0%) profile was more or less a mirror-image of the “decreasing” profile, with interpersonal conflicts starting at the mean level in adolescence, gradually increasing through the years of young adulthood and ending up at a high 2.0 interpersonal conflicts categories above the average level at age 42. Genders were different in their relative proportions in the latent profile groups: males were more likely to be classified to the “steady low” profile compared to females, who were relatively more often classified as belonging to the “decreasing” and “increasing” profile groups (Fig 1). Frequencies of different interpersonal conflicts and events by profile groups are given in S1 Table. While the frequencies of some interpersonal conflict (e.g. conflicts in intimate relationship) were more common than others, the relative differences in these frequencies between profile groups were all concordant with the group solution in general, and no one of the conflict categories was indicated to be a sole driver or otherwise overtly dominant in the group solution.

The estimation and results of the self-esteem latent growth trajectories have been presented earlier [17]. In brief, the best and acceptable model fit was achieved with a non-linear growth model where the last time loading of the slope factor was estimated freely from the data: the self-esteem trajectory grew linearly from age 16 to 32, but after that there was no growth (negative or positive) in it between ages 32 and 42. Here, estimated means of the self-esteem growth factors for a multigroup (females, males) model are given in Table 2 (Total column). The means of the slope were positive indicating that self-esteem grew positively during the study period. Males had a higher self-esteem at age 16 than females (p<0.001). Moreover, females had a faster growth rate (p<0.01) while still having a lower self-esteem level at age 42 (p<0.001).

**Table 2:** Estimated means of the self-esteem latent growth curve factors by gender and interpersonal conflict profile.

		Interpersonal conflict profile group^b^			Group differences^c^
	Total^a^	Steady low	Decreasing	Increasing	
Self-esteem growth factor	Mean(SD)	Mean(SD)	Mean(SD)	Mean(SD)	p (Δχ^2^, df = 1)
***Females***					
Level, age 16	3.45(0.51)	3.49(0.48)	3.30(0.57)	3.47(0.53)	Low vs. Decr. p<0.001 Decr. vs. Incr. p<0.05
Slope^d^	0.25(0.32)	0.25(0.32)	0.31(0.33)	0.15(0.23)	Low vs. Incr. p<0.05 Decr. vs. Incr. p<0.01
Level, age 42^e^	3.85(0.59)	3.90(0.60)	3.82(0.60)	3.73(0.58)	Low vs. Incr. p<0.01
***Males***					
Level, age 16	3.82(0.44)	3.86(0.44)	3.63(0.43)	3.68(0.33)	Low vs. Decr. p<0.001 Low vs. Incr. p<0.01
Slope^d^	0.18(0.31)	0.20(0.28)	0.21(0.34)	0.05(0.29)	Low vs. Incr. p<0.05 Decr. vs. Incr. p<0.05
Level, age 42^e^	4.10(0.58)	4.20(0.53)	3.98(0.65)	3.76(0.63)	Low vs. Decr. p<0.01 Low vs. Incr. p<0.001 Decr. vs. Incr. p<0.05

^a^ Estimates from a multigroup model (two groups: gender), model fit: χ^2^ = 32.6 (df = 9), p<0.001, CFI = 0.98, TLI = 0.98,RMSEA = 0.049

^b^ Estimates from a multigroup model (six groups: gender x interpersonal conflict profile), model fit: χ^2^ = 67.5 (df = 29), p<0.001, CFI = 0.97,TLI = 0.97, RMSEA = 0.060

^c^ Only statistically significant differences are reported.

^d^ Slope represents increases per 10 years.

^e^ From a model where the zero time point was set to the last panel wave at age 42.

**Fig 2:**
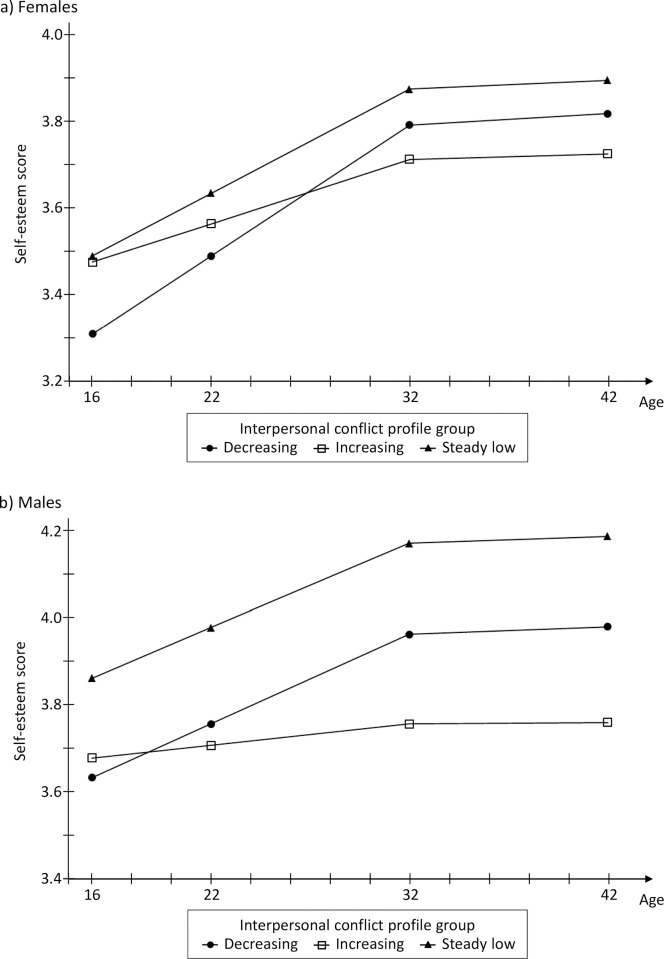
**Self-esteem growth curves in different interpersonal conflict profile groups for females (a) and males (b).**

The means of the latent growth factors of the self-esteem trajectory are given in Table 2 for each interpersonal conflict profile, and the estimated trajectories are plotted in Fig 2. Compared to the other two profiles, the self-esteem trajectory in the “steady low” interpersonal conflict profile group followed a higher path throughout the study period among both genders. The “decreasing” profile had a lower self-esteem level at age 16 compared to the “steady low” group, although the growth rate was of a similar size. Among females, the growth rate in the “decreasing” group was even slightly faster than in the “low” group and the group difference in the self-esteem level was no longer significant at age 42, whereas among males it remained significant. The group with an increasing number of interpersonal conflicts had a significantly slower self-esteem growth rate than the other groups, and also had the lowest self-esteem level at the end of the study period. Females of this profile group had a self-esteem level comparable to the “steady low” group at age 16, while among males this “increase” interpersonal conflict profile had a significantly lower self-esteem level already at the beginning of the study period. While differences between interpersonal conflict profiles in self-esteem trajectories seemed somewhat more pronounced among males, there were only few statistically significant gender differences in these effects: differences between the “increase” and “steady low” profiles in the initial (p<0.05) and end levels (p<0.01) of self-esteem were larger among males compared to females; also the difference in the end level at age 42 between the “decrease” and “steady low” groups was marginally significantly different between genders (p = 0.055).

**Fig 3:**
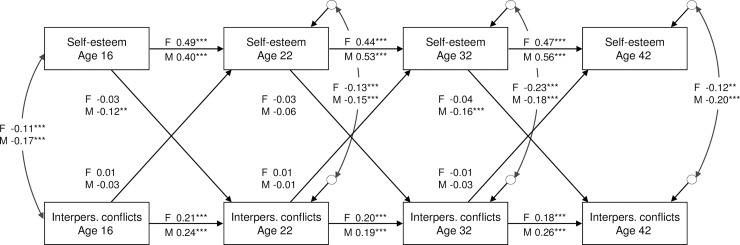
Autoregressive cross-lagged model of self-esteem and interpersonal conflicts. Standardized estimates from a multigroup model: females (above), males (below). Model fit: χ^2^ = 11.99 (df = 12), p = 0.446, CFI = 1.00, TLI = 1.00, RMSEA = 0.000. Note: Autoregressive effects from age 16 to ages 32 and 42 and from age 22 to age 42 were also included in the model, not shown.

The standardized regression estimates of the ARCL model of self-esteem and interpersonal conflicts are given in Fig 3. The autoregressive effects varied between 0.40 and 0.56 for self-esteem and 0.18 and 0.26 for interpersonal conflicts. Cross-sectional correlations between self-esteem and interpersonal conflicts were between -0.11 and -0.23 and significant (for age 22, 32 and 42 study waves correlations are between disturbance terms). Significant lagged effects were found from low self-esteem to later interpersonal conflicts among males from age 16 to 22 and from age 32 to 42 years. Among females, no similar effects were found and the gender differences regarding both of these effects were marginally significant (p = 0.07 and p = 0.05). There were no significant lagged effects from interpersonal conflicts to later self-esteem among either gender.

## Discussion

The present study explored the association between self-esteem and interpersonal conflicts in a longitudinal setting from adolescence to mid-adulthood. Our earlier study had shown that self-esteem develops favorably from adolescence to mid-adulthood in this cohort [17], while the contribution of the present paper was to examine whether interpersonal conflicts have any effect on this “normative” trajectory. As we expected, the developmental trajectory of self-esteem varied according to how interpersonal conflicts were clustered within persons and across time: those who report more interpersonal conflicts and problems had a lower self-esteem level, and those with an increasing number of such conflicts had a slower growth rate in their trajectory. As to the bidirectionality of the effects between self-esteem and interpersonal conflicts, we found no support to suggest that interpersonal conflicts affect later self-esteem, whereas we found some support to suggest that lower self-esteem is related to reporting more interpersonal conflicts later, albeit these effects were relatively weak and present among males only. In general, however, there were only few significant gender differences in the associations between self-esteem and interpersonal conflicts.

Although the identification of interpersonal conflict profiles was not the primary aim of our study and the latent profile method was used principally as an aid to group individuals, the distinctively different longitudinal profiles of interpersonal conflicts we found in our data needs to be noted. While for the large majority interpersonal conflicts do not pile up to any considerable extent, there seem to be groups facing an “excessive” number of interpersonal conflicts comprising many interpersonal contexts at different phases in their life course. When we then compared the developmental growth trajectories of self-esteem between these longitudinal interpersonal conflict profiles, we found–as expected–clear differences between the profile groups: those with more conflicts had a lower and less favorably developing self-esteem trajectory. Especially, those with an increasing number of interpersonal conflicts had a significantly slower growth rate in self-esteem compared to the other two profile groups, and subsequently end up having the poorest self-esteem by mid-adulthood. The result is theoretically sound given the interpersonal nature of self-esteem described in many of its theoretical accounts including the sociometer theory [1,8], classical theories of symbolic interactionism [3,4], the interpersonal theory [36] and others [37,38]. In light of these theories it is perfectly understandable that those with more interpersonal problems and conflicts have lower self-esteem and that increasing number of conflicts is associated with less favorably developing self-esteem trajectory (as compared to the normative development). The result is also well in line with the previous empirical studies that show self-esteem to be responsive to interpersonal events, especially events that implicate rejection [1] (see also [39] for more mixed results). However, as self-esteem also affects the way we perceive our interpersonal relations [7,40], there is another way of looking at these results: it might be that those with an increasing self-esteem trajectory come to see their interpersonal relations as less complicated and with less conflicts as they age and their self-esteem rises; perhaps interpersonal events that appeared devastating in adolescence seem less dramatic when experienced as an adult and a person with a higher self-esteem.

While our results seem sound in the light of theories and previous research, the associations studied here have not been demonstrated in a similar longitudinal setting before. In fact, our results are quite different when compared for example to those reported by Orth and colleagues [18], insofar as social support, marital status and relationship satisfaction had no effect in the self-esteem trajectory throughout the life course in their study. Although differences in the results may be due to differences in the measurements, follow-up times and studied age groups, they may also be due to differences in the analytical methods used: as we used longitudinal profiles of interpersonal measures we were able to identify groups that deviated distinctively from the “normative” group, which likely makes the studied effects more pronounced compared to methods based on total group level means. Whatever the reason, the results of our study suggest that the developmental trajectories of self-esteem and the way interpersonal conflicts are clustered during the life course are closely related.

We also found that the group with a high number of interpersonal conflicts in adolescence, but with a decreasing conflicts profile thereafter, had a self-esteem trajectory that not only started at a lower level in adolescence, but also followed a lower path during the whole study period compared to the group with steady low interpersonal conflicts. The growth rates of the self-esteem trajectories between the two profiles were of the same size. What is interesting here then, is that there seems to be a group of adolescents with many interpersonal conflicts and poor self-esteem, which never really catches up with the good self-esteem level of the “normative” low interpersonal conflict group, even though their number of interpersonal conflicts gradually diminished by age. This result is in line with a study among young adults followed from age 18 to 25 years, where conflicts with parents were found to be associated with a lower initial level, but not with the slope of the self-esteem trajectory [14]. While the effects in our study seemed somewhat more pronounced among males, they nevertheless highlight the importance of adolescence as a developmental phase, since difficulties in adolescence may also have enduring long-term effects on adulthood well-being [41,42]. In this regard, the result also resembles the findings of our earlier paper [17] where the levels of some adolescent covariates (e.g. parental divorce among females) led to constantly equidistant self-esteem trajectories, indicating persistent differences up to mid-adulthood years. These types of enduring associations may reflect some unwanted disparities and the studied covariates—in this case excessive interpersonal conflicts—thus reveal points for possible effective intervention efforts.

Among males, the increasing interpersonal conflict profile had a self-esteem trajectory that had a lower initial level already in adolescence compared to the low interpersonal conflicts group. A similar effect was not observed among females. Although we do not know how self-esteem and interpersonal conflicts were related in that group before age 16, this pattern of findings could suggest that the lower self-esteem in adolescence in this profile group is in part inducing the later increasing number of interpersonal conflicts during adulthood. This interpretation would be in line with the results from the autoregressive models where, again among males only, low self-esteem was predictive of later interpersonal conflicts. As such, the result of low self-esteem inducing later interpersonal conflicts and good self-esteem leading to more satisfying relationships is in line with earlier findings in the literature [19,20,43]. In part this is because self-esteem provides a filter through which we view and respond to the behavior of others [7,40]. Those with low self-esteem do not seem to have the confidence to rely on and accept the positive views others have of them [44]. Instead, they are more sensitized to perceive cues of rejection in their relationship partners and also more ready to react to these cues in such self-protecting ways that in turn increases the likelihood of problematic interactions and conflicts [45,46]. On the other hand, those with more positive self-view have a higher threshold for detecting threats of rejection, they perceive and focus more on those thing that confirms the strength of the relationship and in contrast to those with low self-esteem respond to feeling hurt by drawing closer to their partner [44,45].

However, we have no simple answer as to why we found these cross-lagged effects only among males, especially since our results should have been more in line with previous studies showing that self-concept and self-esteem are more linked to interpersonal relationships and significant others among women than among men [47]. In the study by Kahle et al. [12], the effect of low self-esteem on interpersonal problems was demonstrated among boys. However, gender differences could not be assessed since there were no females in the study. Perhaps poor self-esteem among males is more culturally deviant, e.g. a lack of self-confidence is non-normative for males [48], which makes them more susceptible to defensive and compensatory interaction patterns and behaviors, which in turn leads to conflicts and more problematic relationships [7]. One explanation may also be that as females are likely to be more sensitive to interpersonal events [11], they might also report them more easily, i.e. reporting also milder events that males typically would not report [49]. If this was the case—and in fact in our data females reported more interpersonal conflicts (see Table 1)—the gender differences in the effects between self-esteem and interpersonal conflicts might then be partly due to differences in the severity of the reported conflicts.

Only a few cross-lagged effects were found in the ARCL models; these were modest in size and found only among males. Based on these results, it is difficult to draw any strong conclusions as to the directionality of effects between interpersonal conflicts and self-esteem. That low self-esteem induces later interpersonal problems (and not the other way around) has been reported earlier [12]. A similar result relating to relationship satisfaction and self-esteem was also found by Orth et al. [19]. And as already noted, it is in many ways understandable that low self-esteem leads to interpersonal problems and high self-esteem to closer and rewarding relationships [7,44,45]. However, based both on these accounts, but especially on the sociometer theory and the results supporting it [1], we would have expected the effects to be more bidirectional. While there have also been mixed results relating to the effects of rejection on self-esteem [39], the more probable explanation for our results not being supportive of the sociometer theory relies on the long time intervals between the panel waves. The sociometer should be reflective of rejection and acceptance relatively soon after the events relevant for one’s relational value have taken place. The long-term or life-span effects on the other hand are more likely to develop due to chronic or repeated social rejection [39]. However, this is a type of phenomenon that the cross-lagged analyses with the ten-year intervals are not very sensitive to detect. This is because the longitudinal profiles of interpersonal conflicts as well as the self-esteem trajectories, i.e. the individual level, remain hidden for these types of analyses that are based on total group level means. It is also worth noting that as our measure was of interpersonal conflicts and not of actual rejection or exclusion per se, the results can be linked to the sociometer theory only indirectly, and thus are not directly supportive or unsupportive of it. Nevertheless, regarding the ever ongoing debate as to whether self-esteem is a mere epiphenomenon or whether it predicts other important life outcomes [50–53], our results, even if modest, support more the latter point of view.

### Strengths and limitations

One of the strengths of this study is the practically complete sample of the targeted cohort in the beginning of the study. Higher attrition rates were observed in the later waves of the study, but still only some 13% of the cases provided no follow-up data after the baseline, and in effect an average of 3 (out of 4) instances of measurements were available per case. Attrition was related to male gender and poorer school performance at age 16 [23], but not to self-esteem or the number of interpersonal conflicts at age 16. Nevertheless, all analyses were made using FIML estimation, which retains all cases and uses all available information in the analyzed data set in the parameter estimation, producing less biased estimates than more conventional methods of dealing with missing data [33]. In addition, as school performance at age 16 was related to attrition, it was used as an auxiliary variable in the FIML estimation to increase the plausibility of the MAR assumption. Moreover, some sensitivity analyses were run by leaving out those 272 cases with no follow-up data: the analyses without this group produced essentially the same results regarding the LLPA group solution of interpersonal conflicts, the self-esteem growth trajectories and the associations between these two.

The long follow-up time of 26 years covering developmentally different phases from adolescence to mid-adulthood is one of the strengths of our study. To have only four measurement points covering a period this long is less than optimal, however, and having more measurement points would have provided a more detailed picture of the change trajectories of self-esteem as well as the interpersonal conflict profiles. Regarding the ARCL models, it should be noted that the time lag between the panel waves was not constant throughout the study period (first 6 years, then 10 years) and also that the interpersonal conflicts measures were slightly different between the study waves, thus the estimates between waves are not exactly fully comparable, and comparisons should be made with caution.

The relatively modest effects in the ARCL models may be due to the long time intervals, but also due to possible variation in measurements between the study waves. As we used observed (i.e. not latent) variables, measurement invariance was not studied. Some studies (although with study populations not fully comparable to ours) have indicated good measurement invariance for the Rosenberg self-esteem scale [20,54]. The use of observed (or manifest) variables is also one possible reason to the modest effects in the ARCL models–latent variables would have reflected the true effects more accurately, possibly producing stronger associations. Although ARCL models can be used to study the directionality between effects, this does not mean that causal inferences can be made on these data, especially as some possibly relevant third variables were not studied here, including depression [55], rejection sensitivity [46] and attachment styles [9].

The measure of interpersonal conflicts was rather crude and it covered only the past year in each study wave, not the entire follow-up period. The interpersonal conflict categories included were also quite heterogeneous in their probable significance (persons involved) and duration. To address the questions of self-esteem and interpersonal problems more thoroughly, a more refined measure with a broader scope of interpersonal issues should prove useful (see e.g. Inventory of Interpersonal Problems [56]). Also a measure with items relating to rejection and exclusion would enable more direct link to hypotheses derived from e.g. the sociometer theory. Importantly, it would be advisable to use objective measures of interpersonal problems/conflicts instead of relying solely on self-report. This is especially true in studies on self-esteem, as self-esteem may affect (inflate) the way people perceive and report different socially desirable or undesirable qualities [50]. Using a self-report measure of interpersonal conflicts is thus a limitation in the present study and needs to be kept in mind when interpreting the results.

We used latent profile analyses to group individuals according to their longitudinal patterns of interpersonal conflicts. These types of latent profiles or classes depend always on the given data set and the specified model, and they should not be considered as “real” entities as such, especially as there are no definite agreed-upon rules on how to choose between the group solutions, i.e. the number of latent profiles/classes [31]. Also the differences between the groups of decreasing and increasing interpersonal conflict profiles may not be only quantitative in nature, but also qualitative in that the interpersonal contexts are in part different in different phases of life.

## Conclusions

Our results show that the way interpersonal conflicts cluster within persons and across time from adolescence to adulthood is associated with the way a person’s self-esteem develops during that same period: in essence, those reporting more and an increasing number of interpersonal conflicts have a lower and more slowly developing self-esteem trajectory. This is a theoretically sound result given the core interpersonal nature of self-esteem, although it has rarely been demonstrated in a similar longitudinal setting before. As our measure of interpersonal conflicts was based on subjective perception of such problems, our results may also in part reflect the differences between high and low self-esteem individuals in willingness to report or being tuned to recognize conflicts in their interpersonal relationships. While the question of the directionality of effects between self-esteem and interpersonal conflicts remains somewhat open in the face of the results of this study, it is nevertheless apparent that those with low self-esteem as well as those with an increased number of interpersonal conflicts should be recognized as risk groups probably benefiting from adequate psychosocial interventions.

## Supporting Information

S1 Table Frequencies (%) of interpersonal conflicts by interpersonal conflict profile group. (DOC)

## References

[1] Leary MR. Sociometer theory and the pursuit of relational value: Getting to the root of self-esteem. Eur Rev Soc Psychol. 2005; 16: 75–111.

[2] Baldwin MW, Baccus JR. Maintaining a focus on the social goals underlying self-conscious emotions. Psychol Inq. 2004; 15: 139–144.

[3] Cooley CH. Human nature and the social order New York: Charles Scribner's Sons; 1902.

[4] Mead GH. Mind, self, and society from the standpoint of a social behaviorist Chicago: University of Chicago Press; 1934.

[5] Bowlby J. Attachment and loss: Vol. 1 Attachment. New York: Basic Books; 1969.

[6] Gorrese A, Ruggieri R. Peer attachment and self-esteem: A meta-analytic review. Pers Individ Dif. 2013; 55: 559–568.

[7] Baldwin MW. Self–esteem and close relationship dynamics In: Kernis MH, editor. Self-esteem issues and answers New York: Taylor & Francis; 2006 pp. 359–366.

[8] Leary MR, Baumeister RF. The nature and function of self-esteem: Sociometer theory. Advances in experimental social psychology. 2000; 32: 1–62.

[9] Hankin BL, Kassel JD, Abela JR. Adult attachment dimensions and specificity of emotional distress symptoms: prospective investigations of cognitive risk and interpersonal stress generation as mediating mechanisms. Pers Soc Psychol Bull. 2005; 31: 136–151. 10.1177/0146167204271324 15574668

[10] Wright AG, Hallquist MN, Beeney JE, Pilkonis PA. Borderline personality pathology and the stability of interpersonal problems. J Abnorm Psychol. 2013; 122: 1094–1100. 10.1037/a0034658 24364612 PMC3874134

[11] Liu RT, Alloy LB. Stress generation in depression: A systematic review of the empirical literature and recommendations for future study. Clin Psychol Rev. 2010; 30: 582–593. 10.1016/j.cpr.2010.04.010 20478648 PMC3049314

[12] Kahle LR, Kulka RA, Klingel DM. Low adolescent self-esteem leads to multiple interpersonal problems: A test of social-adaptation theory. J Pers Soc Psychol. 1980; 39: 496–502. 7431206 10.1037//0022-3514.39.3.496

[13] Donnellan MB, Trzesniewski KH, Robins RW, Moffitt TE, Caspi A. Low self-esteem is related to aggression, antisocial behavior, and delinquency. Psychol Sci. 2005; 16: 328–335. 10.1111/j.0956-7976.2005.01535.x 15828981

[14] Galambos NL, Barker ET, Krahn HJ. Depression, self-esteem, and anger in emerging adulthood: seven-year trajectories. Dev Psychol. 2006; 42: 350–365. 10.1037/0012-1649.42.2.350 16569173

[15] Greene ML, Way N. Self-esteem trajectories among ethnic minority adolescents: A growth curve analysis of the patterns and predictors of change. J Res Adolesc. 2005; 15: 151–178.

[16] Birkeland MS, Melkevik O, Holsen I, Wold B. Trajectories of global self-esteem development during adolescence. J Adolesc. 2012; 35: 43–54. 10.1016/j.adolescence.2011.06.006 21764114

[17] Kiviruusu O, Huurre T, Aro H, Marttunen M, Haukkala A. Self-esteem growth trajectory from adolescence to mid-adulthood and its predictors in adolescence. Adv Life Course Res. 2015; 23: 29–43. 10.1016/j.alcr.2014.12.003 26047839

[18] Orth U, Trzesniewski KH, Robins RW. Self-esteem development from young adulthood to old age: a cohort-sequential longitudinal study. J Pers Soc Psychol. 2010; 98: 645–658. 10.1037/a0018769 20307135

[19] Orth U, Robins RW, Widaman KF. Life-span development of self-esteem and its effects on important life outcomes. J Pers Soc Psychol. 2012; 102: 1271–1288. 10.1037/a0025558 21942279

[20] Orth U, Maes J, Schmitt M. Self-esteem development across the life span: A longitudinal study with a large sample from Germany. Dev Psychol. 2015; 51: 248–259. 10.1037/a0038481 25485608

[21] Trzesniewski KH, Donnellan MB, Moffitt TE, Robins RW, Poulton R, Caspi A. Low self-esteem during adolescence predicts poor health, criminal behavior, and limited economic prospects during adulthood. Dev Psychol. 2006; 42: 381–390. 10.1037/0012-1649.42.2.381 16569175

[22] Kling K, Hyde J, Showers C, Buswell B. Gender differences in self-esteem: A meta-analysis. Psychol Bull. 1999; 125: 470–500. 10414226 10.1037/0033-2909.125.4.470

[23] Eerola M, Huurre T, Aro H. The problem of attrition in a Finnish longitudinal survey on depression. Eur J Epidemiol. 2005; 20: 113–120. 15756911 10.1007/s10654-004-1657-0

[24] Aro H. Parental discord, divorce and adolescent development. Eur Arch Psychiatry Neurol Sci. 1988; 237: 106–111. 3360023 10.1007/BF00382374

[25] Rosenberg M. Society and the adolescent self-image. Princeton (NJ): Princeton University Press; 1965.

[26] Aro H. Life stress and psychosomatic symptoms among 14 to 16-year old Finnish adolescents. Psychol Med. 1987; 17: 191–201. 3575572 10.1017/s0033291700013088

[27] Huurre T, Junkkari H, Aro H. Long–term Psychosocial effects of parental divorce. Eur Arch Psychiatry Clin Neurosci. 2006; 256: 256–263. 10.1007/s00406-006-0641-y 16502211

[28] Muthén LK, Muthén BO. Mplus User’s Guide Los Angeles: Muthén and Muthén; 1998–2012.

[29] Cole VT. Modeling complex longitudinal data from heterogeneous samples using longitudinal latent profile analysis. M.A. Thesis, University of North Carolina at Chapel Hill. 2014. Available: https://cdr.lib.unc.edu/record/uuid:a400e33e-7efa-417a-adaa-2a95d6510663.

[30] Gibson WA. Three multivariate models: Factor analysis, latent structure analysis, and latent profile analysis. Psychometrika. 1959; 24: 229–252.

[31] Nylund KL, Asparouhov T, Muthén BO. Deciding on the number of classes in latent class analysis and growth mixture modeling: A Monte Carlo simulation study. Struct Equ Modeling. 2007; 14: 535–569.

[32] Bollen KA, Curran PJ. Latent curve models: A structural equation perspective. Hoboken (NJ): John Wiley & Sons; 2006.

[33] Widaman KF III. Missing data: What to do with or without them. Monogr Soc Res Child Dev. 2006; 71: 42–64.

[34] Allison PD. Missing data techniques for structural equation modeling. J Abnorm Psychol. 2003; 112: 545–557. 10.1037/0021-843X.112.4.545 14674868

[35] Hu L, Bentler PM. Cutoff criteria for fit indexes in covariance structure analysis: Conventional criteria versus new alternatives. Struct Equ Modeling. 1999; 6: 1–55.

[36] Safran JD. Towards a refinement of cognitive therapy in light of interpersonal theory: I. Theory. Clin Psychol Rev. 1990; 10: 87–105.

[37] Baldwin MW, Sinclair L. Self-esteem and" if… then" contingencies of interpersonal acceptance. J Pers Soc Psychol. 1996; 71: 1130–1141. 8979382 10.1037//0022-3514.71.6.1130

[38] Pyszczynski T, Greenberg J, Solomon S, Arndt J, Schimel J. Why do people need self-esteem? A theoretical and empirical review. Psychol Bull. 2004; 130: 435–468. 10.1037/0033-2909.130.3.435 15122930

[39] Blackhart GC, Nelson BC, Knowles ML, Baumeister RF. Rejection elicits emotional reactions but neither causes immediate distress nor lowers self-esteem: a meta-analytic review of 192 studies on social exclusion. Pers Soc Psychol Rev. 2009; 13: 269–309. 10.1177/1088868309346065 19770347

[40] Wallace HM, Tice DM. Reflected appraisal through a 21st-century looking glass In: Leary MR, Tangney JP, editors. Handbook of self and identity. 2nd ed. New York, NY, USA: The Guilford Press; 2012 pp. 124–140.

[41] Schulenberg JE, Maggs JL, O’Malley PM. How and why the understanding of developmental continuity and discontinuity is important. The sample case of long-term consequences of adolescent substance use In: Mortimer JT, Shanahan MJ, editors. Handbook of the Life Course New York: Kluwer Academic/Plenum Publishers; 2003 pp. 413–436.

[42] Johnson MK, Crosnoe R, Elder GH. Insights on adolescence from a life course perspective. J Res Adolesc. 2011; 21: 273–280. 10.1111/j.1532-7795.2010.00728.x 21483644 PMC3072576

[43] Johnson MD, Galambos NL, Krahn HJ. Self-esteem trajectories across 25 years and midlife intimate relations. Pers Relatsh. 2015; 22: 635–646.

[44] Murray SL, Holmes JG, Griffin DW. Self-esteem and the quest for felt security: how perceived regard regulates attachment processes. J Pers Soc Psychol. 2000; 78: 478–498. 10743875 10.1037//0022-3514.78.3.478

[45] Murray SL, Bellavia GM, Rose P, Griffin DW. Once hurt, twice hurtful: how perceived regard regulates daily marital interactions. J Pers Soc Psychol. 2003; 84: 126–147. 12518975

[46] Downey G, Feldman SI. Implications of rejection sensitivity for intimate relationships. J Pers Soc Psychol. 1996; 70: 1327–1343. 8667172 10.1037//0022-3514.70.6.1327

[47] Josephs RA, Markus HR, Tafarodi RW. Gender and self-esteem. J Pers Soc Psychol. 1992; 63: 391–402. 1403622 10.1037//0022-3514.63.3.391

[48] Wood W, Eagly AH. Two traditions of research on gender identity. Sex Roles. 2015; 73: 461–473.

[49] Jenkins SR, Goodness K, Buhrmester D. Gender differences in early adolescents’ relationship qualities, self-efficacy, and depression symptoms. J Early Adolesc. 2002; 22: 277–309.

[50] Baumeister RF, Campbell JD, Krueger JI, Vohs KD. Does High Self-Esteem Cause Better Performance, Interpersonal Success, Happiness, or Healthier Lifestyles? Psychol Sci Public Interest. 2003; 4: 1–44. 10.1111/1529-1006.01431 26151640

[51] Dubois DL, Tevendale HD. Self-esteem in childhood and adolescence: Vaccine or epiphenomenon? Appl Prev Psychol. 1999; 8: 103–117.

[52] Orth U, Robins RW. The development of self-esteem. Curr Dir Psychol Sci. 2014; 23: 381–387.

[53] Swann WB Jr, Chang-Schneider C, Larsen McClarty K. Do people's self-views matter? Self-concept and self-esteem in everyday life. Am Psychol. 2007; 62: 84–94. 10.1037/0003-066X.62.2.84 17324034

[54] Gana K, Saada Y, Bailly N, Joulain M, Hervé C, Alaphilippe D. Longitudinal factorial invariance of the Rosenberg Self-Esteem Scale: Determining the nature of method effects due to item wording. Journal of Research in Personality. 2013; 47: 406–416.

[55] Sowislo JF, Orth U. Does low self-esteem predict depression and anxiety? A meta-analysis of longitudinal studies. Psychol Bull. 2013; 139: 213 10.1037/a0028931 22730921

[56] Horowitz LM, Rosenberg SE, Baer BA, Ureño G, Villaseñor VS. Inventory of interpersonal problems: psychometric properties and clinical applications. J Consult Clin Psychol. 1988; 56: 885–892. 3204198 10.1037//0022-006x.56.6.885

