# Supplementary material for: Interpersonal Conflicts and Development of Self-Esteem from Adolescence to Mid-Adulthood. A 26-Year Follow-Up
Source: PLoS One. 2016 Oct 18;11(10):e0164942. doi: 10.1371/journal.pone.0164942 (PMC5068799; doi:10.1371/journal.pone.0164942)
Supplement: S1 Table — (DOC) [file pone.0164942.s001.doc]

**S1 Table. Frequencies (%) of interpersonal conflicts by interpersonal conflict prof**ile group.

|  |  | Females | | | |  | Males | | | |  | Total | | | |  |
| --- | --- | --- | --- | --- | --- | --- | --- | --- | --- | --- | --- | --- | --- | --- | --- | --- |
| Interpersonal event |  | Low | Decr. | Incr. | p |  | Low | Decr. | Incr. | p |  | Low | Decr. | Incr. | p |  |
| Age 16 |  |  |  |  |  |  |  |  |  |  |  |  |  |  |  |  |
| Break-up with girl/boyfriend |  | 18 | 73 | 29 | <.001 |  | 14 | 58 | 24 | <.001 |  | 16 | 67 | 27 | <.001 |  |
| Conflicts with mother |  | 6 | 70 | 17 | <.001 |  | 4 | 61 | 10 | <.001 |  | 5 | 66 | 14 | <.001 |  |
| Conflicts with father |  | 5 | 53 | 12 | <.001 |  | 3 | 54 | 8 | <.001 |  | 4 | 53 | 11 | <.001 |  |
| Conflicts with teacher |  | 3 | 35 | 5 | <.001 |  | 7 | 59 | 10 | <.001 |  | 5 | 45 | 7 | <.001 |  |
| Conflicts with classmates |  | 2 | 19 | 7 | <.001 |  | 2 | 16 | 4 | <.001 |  | 2 | 18 | 6 | <.001 |  |
| Age 22 |  |  |  |  |  |  |  |  |  |  |  |  |  |  |  |  |
| Break-up with girl/boyfriend |  | 21 | 30 | 28 | .022 |  | 26 | 32 | 30 | .374 |  | 23 | 31 | 29 | .022 |  |
| Separation/divorce |  | 5 | 11 | 8 | .023 |  | 3 | 4 | 9 | .014 |  | 4 | 8 | 8 | .001 |  |
| Conflicts with teacher/superior |  | 3 | 8 | 13 | <.001 |  | 4 | 18 | 13 | <.001 |  | 4 | 12 | 13 | <.001 |  |
| Conflicts with mother |  | 4 | 12 | 10 | <.001 |  | 5 | 18 | 18 | <.001 |  | 5 | 14 | 13 | <.001 |  |
| Conflicts with father |  | 3 | 9 | 8 | .003 |  | 5 | 20 | 11 | <.001 |  | 4 | 13 | 9 | <.001 |  |
| Conflicts in intimate relationship |  | 31 | 54 | 61 | <.001 |  | 30 | 47 | 48 | <.001 |  | 31 | 51 | 56 | <.001 |  |
| Conflicts with friends |  | 13 | 26 | 28 | <.001 |  | 13 | 23 | 38 | <.001 |  | 13 | 25 | 31 | <.001 |  |
| Conflicts with colleagues/fellow students |  | 12 | 21 | 24 | <.001 |  | 10 | 13 | 28 | <.001 |  | 11 | 18 | 25 | <.001 |  |
| Age 32 |  |  |  |  |  |  |  |  |  |  |  |  |  |  |  |  |
| Break-up with girl/boyfriend |  | 7 | 11 | 14 | .009 |  | 8 | 12 | 24 | <.001 |  | 7 | 12 | 18 | <.001 |  |
| Separation/divorce |  | 4 | 8 | 8 | .073 |  | 4 | 3 | 13 | .003 |  | 4 | 6 | 10 | .003 |  |
| Conflicts with mother |  | 6 | 14 | 16 | <.001 |  | 4 | 7 | 17 | <.001 |  | 5 | 11 | 16 | <.001 |  |
| Conflicts with father |  | 6 | 12 | 14 | .004 |  | 3 | 8 | 15 | <.001 |  | 5 | 11 | 14 | <.001 |  |
| Conflicts in intimate relationship |  | 32 | 55 | 61 | <.001 |  | 31 | 50 | 73 | <.001 |  | 31 | 53 | 66 | <.001 |  |
| Conflicts with friends |  | 10 | 24 | 42 | <.001 |  | 7 | 23 | 40 | <.001 |  | 8 | 24 | 41 | <.001 |  |
| Conflicts with colleagues |  | 14 | 17 | 41 | <.001 |  | 14 | 29 | 49 | <.001 |  | 14 | 22 | 44 | <.001 |  |
| Age 42 |  |  |  |  |  |  |  |  |  |  |  |  |  |  |  |  |
| Break-up with girl/boyfriend |  | 2 | 7 | 32 | <.001 |  | 2 | 6 | 32 | <.001 |  | 2 | 7 | 32 | <.001 |  |
| Separation/divorce |  | 2 | 5 | 22 | <.001 |  | 2 | 1 | 13 | <.001 |  | 2 | 4 | 19 | <.001 |  |
| Conflicts with mother |  | 3 | 10 | 21 | <.001 |  | 1 | 1 | 17 | <.001 |  | 2 | 7 | 19 | <.001 |  |
| Conflicts with father |  | 1 | 4 | 13 | <.001 |  | 1 | 1 | 16 | <.001 |  | 1 | 3 | 14 | <.001 |  |
| Conflicts in intimate relationship |  | 26 | 43 | 80 | <.001 |  | 24 | 37 | 76 | <.001 |  | 25 | 41 | 78 | <.001 |  |
| Conflicts with friends |  | 3 | 10 | 44 | <.001 |  | 3 | 10 | 43 | <.001 |  | 3 | 10 | 43 | <.001 |  |
| Conflicts with colleagues |  | 8 | 14 | 57 | <.001 |  | 7 | 14 | 62 | <.001 |  | 7 | 14 | 59 | <.001 |  |
| Conflicts with superior |  | 7 | 11 | 43 | <.001 |  | 4 | 11 | 55 | <.001 |  | 6 | 11 | 47 | <.001 |  |

Interpersonal problems profile group abbreviations: Low=Steady low; Decr.=Decreasing; Incr.=Increasing; all p-values from chi-square test (df=2).
